# Causal Modeling Using Network Ensemble Simulations of Genetic and Gene Expression Data Predicts Genes Involved in Rheumatoid Arthritis

**DOI:** 10.1371/journal.pcbi.1001105

**Published:** 2011-03-10

**Authors:** Heming Xing, Paul D. McDonagh, Jadwiga Bienkowska, Tanya Cashorali, Karl Runge, Robert E. Miller, Dave DeCaprio, Bruce Church, Ronenn Roubenoff, Iya G. Khalil, John Carulli

**Affiliations:** 1 Gene Network Sciences, Cambridge, Massachusetts, United States of America; 2 Biogen Idec, Cambridge, Massachusetts, United States of America; University of Auckland, New Zealand

## Abstract

Tumor necrosis factor α (TNF-α) is a key regulator of inflammation and rheumatoid arthritis (RA). TNF-α blocker therapies can be very effective for a substantial number of patients, but fail to work in one third of patients who show no or minimal response. It is therefore necessary to discover new molecular intervention points involved in TNF-α blocker treatment of rheumatoid arthritis patients. We describe a data analysis strategy for predicting gene expression measures that are critical for rheumatoid arthritis using a combination of comprehensive genotyping, whole blood gene expression profiles and the component clinical measures of the arthritis Disease Activity Score 28 (DAS28) score. Two separate network ensembles, each comprised of 1024 networks, were built from molecular measures from subjects before and 14 weeks after treatment with TNF-α blocker. The network ensemble built from pre-treated data captures TNF-α dependent mechanistic information, while the ensemble built from data collected under TNF-α blocker treatment captures TNF-α independent mechanisms. *In silico* simulations of targeted, personalized perturbations of gene expression measures from both network ensembles identify transcripts in three broad categories. Firstly, 22 transcripts are identified to have new roles in modulating the DAS28 score; secondly, there are 6 transcripts that could be alternative targets to TNF-α blocker therapies, including CD86 - a component of the signaling axis targeted by Abatacept (CTLA4-Ig), and finally, 59 transcripts that are predicted to modulate the count of tender or swollen joints but not sufficiently enough to have a significant impact on DAS28.

## Author Summary

The collection and analysis of clinical data has played a key role in providing insights into the diagnosis, prognosis and treatment of disease. However, it is imperative that molecular and genetic data also be collected and integrated into the creation of network models, which capture underlying mechanisms of disease and can be interrogated to elucidate previously unknown biology. Bringing data from the clinic to the bench completes the cycle of translational research, which we demonstrate with this work. We built disease models from genetics, whole blood gene expression profiles and the component clinical measures of rheumatoid arthritis using a data-driven approach that leverages supercomputing. Genetic factors can be utilized as a source of perturbation to the system such that causal connections between genetics, molecular entities and clinical outcomes can be inferred. The existing TNF-α blocker treatments for rheumatoid arthritis are only effective for approximately 2/3 of the affected population. We identified novel therapeutic intervention points that may lead to the development of alternatives to TNF-α blocker treatments. We believe this approach will provide improved drug discovery programs, new insights into disease progression, increased drug efficacy and novel biomarkers for chronic and complex diseases.

## Introduction

Rheumatoid arthritis (RA) is a common autoimmune disease that is characterized by inflammation and destruction of the joints [1]. Inflammation is caused by the infiltration of inflammatory cells, including neutrophils, macrophages, B- and T-cells into the normally acellular synovial tissue that lines the junction of bones. A key feature of the inflammatory process is the release by the infiltrating cells of proinflammatory cytokines, including TNF-α, interleukin-1 (IL-1), interleukin-6 (IL-6), and others. These cytokines promote further inflammation and joint destruction by activating infiltrating immune cells as well as resident bone cells and promoting the release of degradative enzymes such as matrix metalloproteases and cathepsins. Another key feature of the disease is the presence of autoantibodies directed against citrullinated proteins and other targets that contribute to joint damage as well as systemic manifestations of RA [2]. Like other autoimmune diseases, RA is caused by complex interactions between genes and environment [3].

RA is treated with small molecule, disease-modifying anti-rheumatic drugs (DMARDs) including Methotrexate, Sulfasalazine, Leflunomide and others [4]. Biologic agents include several tumor necrosis factor alpha (TNF-α) blockers such as Etanercept, Infliximab and Adalimumab, co-stimulation blockers (abatacept or CTLA4-Ig) and B-cell depleters (rituximab). DMARDs are often combined with tumor necrosis factor alpha (TNF-α) blockade. For many patients, TNF-α blockade effectively relieves arthritis symptoms as measured by American College of Rheumatology (ACR) or Disease Activity Score (DAS) scoring systems that measure numbers of tender and swollen joints as well as other clinical parameters. Typically 39% of patients score better than ACR 50 in etanercept trials when dosed at 10 mg or 25 mg twice weekly, and 64% of patients scored better than ACR 20 [5]. However, for particular subsets of rheumatoid arthritis patients, TNF-α blockade does not appear to relieve the symptoms of RA.

A genetic component to RA has been established from twin and family studies. The estimated heritability of RA is about 60%, and the genetic basis is complex with consistent association of *HLA, PTPN22, TRAF1-C5*, and several other loci [6]. Some of these genetic variants have also been associated with differential response to treatment with TNF-α blockers [7]. However, establishing the causal molecular mechanisms by which genetic variants affect RA phenotypes or differential response to TNF-α blocker to inform rational selection of molecular intervention targets for RA remains a challenging problem.

Establishing causal mechanisms, particularly in clinical data, is a difficult exercise and is often assessed using two popular approaches [8]. One approach uses probabilistic evidence from cross-sectional population studies and discovers new stable statistical associations between the measurements. However, discovering statistical links between the measurements alone cannot determine the causal direction. Another uses mechanistic evidence arising from knowledge of an existing physical property and establishes a predictable dependency over time. However, discovering new mechanistic knowledge demands experimentation and leads directly to the first approach of statistical, probabilistic analysis of the collected data.

Probabilistic or mechanistic causality models alone appear to be at once insufficient and irreconcilable. Scientific inferences from approaches that are unable to effectively reconcile these two notions of causality typically experience delays in their acceptance. There have been many instances of strong probabilistic links in clinical data that have not been accepted until the mechanism had been discovered. Rigorous clinical science correctly requires that both probabilistic and mechanistic arguments need to be met simultaneously before a particular claim can be accepted as causal [8].

The scientific method provides a framework to address both aspects of causality simultaneously [8]. First, existing evidence is used to propose a constrained *rational mechanism*. Second, controlled experiments or *perturbations* are designed to test the mechanism, and relevant data are collected on the proposed mechanistic molecules. Finally, an appropriate systematization of the collected data *deduces probabilistic reflections* of known mechanisms and *infers* new aspects of the mechanism that can be directly tested. If the inferred aspects are confirmed, the proposed mechanism is accepted as causal.

To develop a causal data analysis approach to rational drug target and biomarker discovery in RA, we used published data from the Autoimmune Biomarkers Collaborative Network (ABCoN) [9]. The ABCoN recruited more than 100 patients naïve to anti-TNF treatment for systematic clinical and molecular analysis. Clinical data for calculating Disease Activity Score 28 (DAS28), and blood samples for genetic (SNP) and expression profiling analysis, were collected at baseline (pretreatment), as well as 6 weeks and 14 weeks after starting therapy for one of three anti-TNF molecules (Etanercept, Infliximab or Adalimumab). In our analysis of ABCoN data, we assume a rational mechanism that genetic variation arising from meiosis in this study population together with drug therapy are systematic perturbations that impact RA through multiple molecular and physiological interactions that are probabilistically reflected in the blood transcription profile data. Systematization of the gene expression and clinical data enable us to distinguish transcripts that play a role in modulating RA phenotypes from those that are either simply correlated with or secondary to the phenotypes.

Bayesian networks provide a convenient framework for systematizing data to deduce probabilistic orderings and modeling large systems of interacting variables [10], [11], [12], [13], [14]. Most previous studies have concentrated on either estimating the structural connections in the system under study or on the identification of disease associated genetic polymorphisms. Simulation of Bayesian networks can be used to predict the effect of specific interventions. Whereas some previous studies make predictions based on a single network topology [15], [16], [17], [18], [19], our approach adds to these in two important ways. First it generates a statistical sample, or ensemble, of network structures that are consistent with data collected [20], [21]. Second, it enables quantitative prediction of the effects of perturbations [22] that account for uncertainty about network topology.

With the ability to predict the impact of specific interventions that were not part of the collected data in defined study subjects, the ensemble organizes the data into a rational model and also predicts unseen events. In this regard, these simulation-ready integrative genomic ensembles capture the essence of the scientific method described previously.

Here we concentrate on the prediction of gene expression levels that are critical to explaining the number of swollen joints (SJ), the number of tender joints (TJ), the amount of pain and the plasma concentration of C-reactive protein (CRP) in the subjects enrolled in the ABCoN trial with and without TNF-α blocker treatment.

Sets of biomarkers predictive of TNF-α blocker response have been identified [23], [24]. This should serve as an important diagnostic tool to help determine who would or would not benefit from a TNF-α blocker therapy. While there are treatment options for those who do not respond to TNF-α blocker therapies, identifying those patients before they begin biologics or early in their treatment would help rationalize their treatment. Additionally, uncovering molecular alterations underlying TNF-α blocker response will be critical to discovering new and effective therapies for RA.

## Results

### Using ABCoN study data to explore mechanisms of differential response to TNF-α blockade

While DMARDs, TNF-α blockers and other treatments are available for RA patients [1], [25], there is still a need to identify new drug targets for patients whose disease does not respond to available therapies. If the molecular mechanism for TNF-α blocker failure were understood, the knowledge would allow drug researchers to more effectively select molecules that could target this particular subgroup of patients. Simulation-capable ensemble network models of cause and effect are theoretically capable of providing clues to the reasons some patients do not respond.

The ideal strategy to learn the probabilistic mechanisms of non-response would be to integrate circulating drug concentrations (pharmacokinetic data), measures of the effectiveness of the drug (pharmacodynamic data), genetic variation, relevant molecular measures from the disease-affected tissues and finally, components of DAS28 into a single network ensemble model. Differences in all these measures would lead to an ensemble model that could effectively recover TNF-α blockade response in genetically defined subjects. To recover non-response targets, molecular measures for non-responding subjects would be set in the ensemble and systematic *in silico* perturbations for each non-responding subject would be completed to give a set of intervention points for these particular subjects. For alternative targets to TNF-α in non-responding subjects, the pharmacokinetic variables would be set to mimic a TNF-α blocker concentration of zero. If the focus were to find combination therapies, the simulations would be completed where the pharmacokinetic variables mimicked realistic circulating doses.

For the ABCoN study, there are no pharmacokinetic or pharmacodynamic measures, such as phospho-proteomic data available to enable the modeling strategy outlined. In addition, and as seen in this data set and other studies [26], TNF-α transcript levels vary so little in whole blood that they cannot be considered as a surrogate for pharmacokinetic or pharmacodynamic measures. This means that the data cannot be used to effectively understand the probabilistic mechanisms of response, or non-response, to TNF-α blocker therapy.

However, while a mechanistic analysis is difficult within the current study design, we can investigate transcription variation with and without an undefined amount of circulating TNF-α blocker activity with the expectation that models built using data collected before TNF-α blocker therapy will be informative for untreated RA and mainly reflect TNF-α dependent mechanisms.

Additionally, models built using data collected from treated subjects are expected to reveal aspects of RA that are still important even after TNF-α signaling has been interdicted. Critical transcripts identified from the network ensemble built from data collected after treatment could represent both starting points for drug target identification for subjects who will not respond and combination therapy targets that are only important when TNF-α blocker is circulating in the patient.

### Reverse-Engineer and Forward-Simulate (REFS) predictive framework

**Figure 1:**
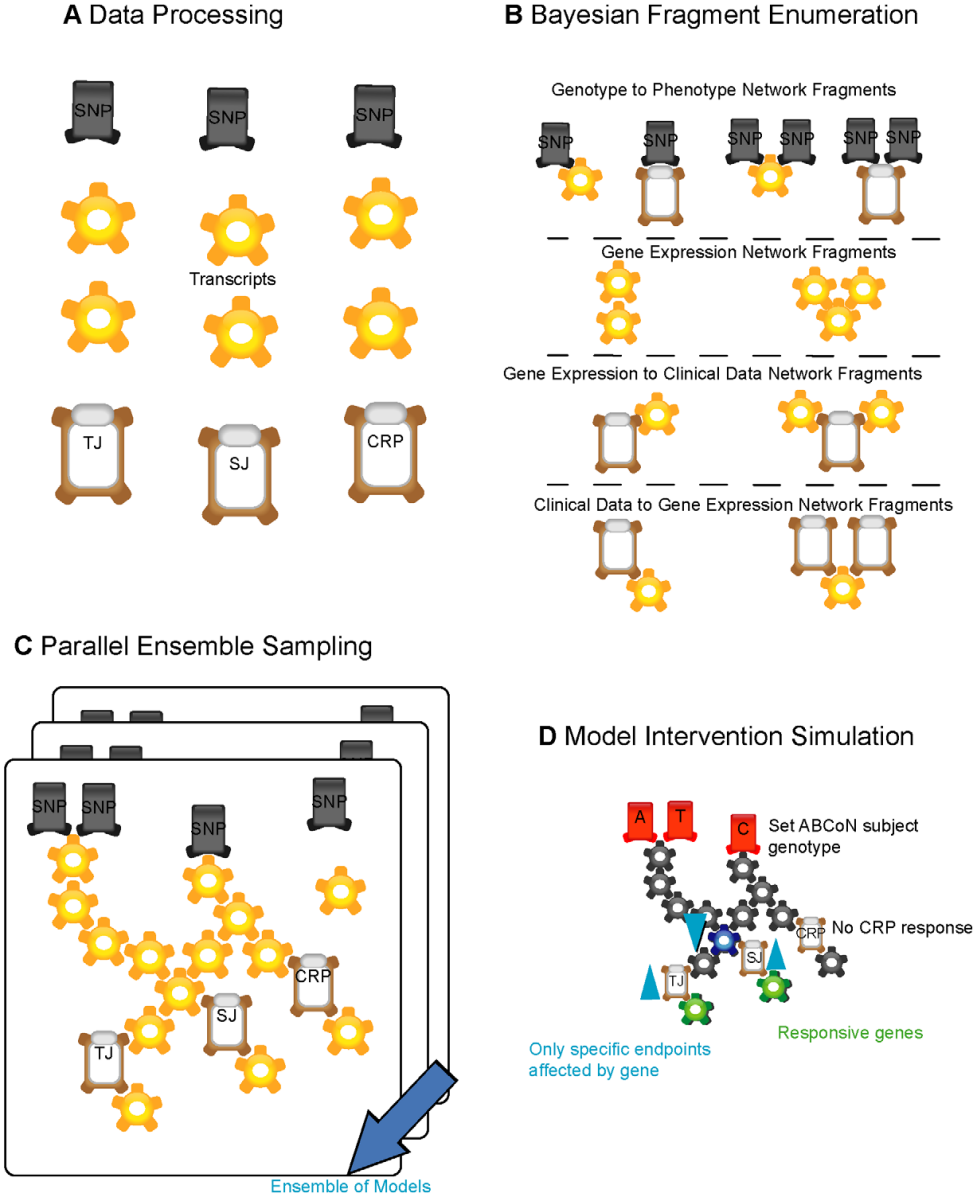
Schematic of Bayesian network reverse engineering and Monte Carlo simulation. A. Genetic, gene expression and phenotypic data are prepared for modeling by formatting followed by investigation to select the appropriate data transformation for the particular data type. Confounding factors and other explanatory variables are considered and modeled if appropriate. B. Likely fragments for network reconstruction are identified by scoring all 2-, 3- and 4-variable combinations with the constraint that SNPs are causally upstream. There are too many scored combinations to consider all during network reconstruction. The fragments that had the most likely Bayesian scores for each individual variable were identified and retained for network reconstruction. C. Parallel global network sampling constructs an ensemble of 1024 network structures that explain the data. The probabilistic directionality computed by the Bayesian framework allows inferences to be made about what lies upstream and downstream of particular phenotypic variables D. Diversity in network structures identified during network reconstruction captures uncertainty in the model. Hypotheses are extracted from the network ensemble by completing Monte Carlo simulations of “what-if” scenarios. Down-regulating the blue transcript would be expected to impact both TJ and SJ, while leaving CRP unchanged. The change in these phenotypic parameters would further predict *reactive* transcription changes in the liver. The separation of upstream and downstream components can identify potential intervention points and markers.

Our method works by considering all possible associations between DNA variation, gene expression, and RA clinical data, resulting in a collection of network fragments derived from these measurements and reflecting not only associations between the traits, but how the different variables are causally associated as well. Each network fragment defines a quantitative, continuous relationship among all possible sets of 3 or fewer measured molecular variables. Because of the experimental design, where DNA variation is leveraged as the randomization mechanism needed to make causal inferences, these fragments approximate stable, probabilistic cause and effect relationships [11]. The relationship is supported by a Bayesian probabilistic score that deduces how likely the candidate relationship is given the measurements and also penalizes the relationship for its mathematical complexity (Figure 1B). By exhaustively scoring all of the possible pairwise and three-way relationships inferred from the DNA, expression, and RA clinical data, the most likely fragments can be identified and held aside preferentially in the collection for later use. Network fragments that include SNPs are constrained such that the genotypes are always upstream of gene expression or clinical outcome data, reflecting the assumption that genotypes are systematic perturbations to the disease. However, network fragments that are comprised of gene expression and clinical outcome data alone could affect each other through multiple causal mechanisms, and therefore we considered all possible orderings of three or fewer variables. In addition to assessing the probability of a particular relationship, the quantitative parameters of the relationship are computed and stored, an important departure from previous methods that have primarily focused on structure. Rather than discarding this quantitative information, we store it so that it can be queried to draw more complex inferences later on in our process.

In the second phase, we estimate an ensemble of network models based on data from the integrative genomics experiment. A statistical definition of the ensemble is a sample of networks drawn from all possible networks consistent with the data, and whose properties are robust, regardless of either the actual structures contained within it or the algorithmic starting conditions that generated it. Even though a normally distributed random variable can take on infinitely many values, sampling even as few as 30 numbers from the distribution can provide reliable estimates of the mean and standard deviation of the distribution, thereby perfectly characterizing the behavior of the random variable. In much the same way, our approach samples from the space of all possible networks to approximate the distribution of that space, enabling an effective characterization of that space. In the third phase we use forward simulation of networks in the ensemble to generate predictions of the effects of perturbations. The entire process is summarized in Figure 1. The entire process is repeated for data collected before and 14 weeks after TNF-α blocker therapy, giving two distinct network ensembles that capture untreated and treated aspects of RA mechanism.

**Figure 2:**
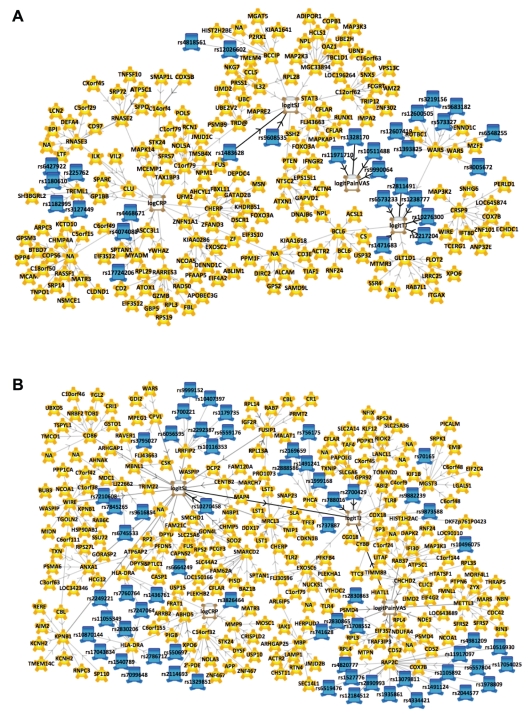
Consensus topology of network ensembles for pre-treated and TNF-α blocker treated data. A. Snapshot of the network ensemble at 2.5% consensus topology generated from pre-treated subjects. Pain does not appear to be controlled by measures extracted from whole blood mRNA profiling in pre-treated subjects. B. Snapshot of the network consensus topology at 2.5% consensus generated from TNF-α blocker treated data.

Summaries of data processing, diagrams of the consensus structure of the two models with respect to the DAS28 components (Figure 2) and the mathematical assessment of the quality of the models generated by the framework are provided (Table 1 in Text S1 and Figures S1–S3). Briefly, the untreated network ensemble was built using 6,075 SNPs, which included imputed genotypes for previously identified SNPs associated with RA, 4,794 gene expression values and 4 DAS28 component scores. The treated network ensemble was built using 6,076 SNPs, 4,512 gene expression values and 4 DAS28 component scores. The network sample properties were assessed by completing three independent network sampling procedures using different, random starting conditions. There were no significant differences in the sample properties regardless of the starting conditions demonstrating that the sampling procedure had converged. The predictive properties of the network sample were assessed using simulations designed to measure how accurate the network ensemble recovered the actual observed data (Text S1).

**Table 1:** Category 1 Transcripts from the post-treatment model.

Gene Symbol	Name	TJ	SJ	DAS28
RAP2C	RAP2C, member of RAS oncogene family	X	X	X
ANXA1	Annexin A1	X	X	X
GON4L	gon-4-like (C. elegans)	X	X	X
CPVL	carboxypeptidase, vitellogenic-like	X	X	X
SMARCD2	SWI/SNF related, matrix associated, actin dependent regulator of chromatin, subfamily d, member 2	X	X	X
SLC6A6	solute carrier family 6 (neurotransmitter transporter, taurine), member 6	X	X	X
EID1	EP300 interacting inhibitor of differentiation 1	X	X	X
MALAT1	metastasis associated lung adenocarcinoma transcript 1 (non-coding RNA)	X	X	X
EIF3D	eukaryotic translation initiation factor 3, subunit D	X		X
CHCHD2	coiled-coil-helix-coiled-coil-helix domain containing 2	X		X
NDE1	nudE nuclear distribution gene E homolog 1 (A. nidulans)	X		X
NCOA1	nuclear receptor coactivator 1	X		X
ARHGAP25	Rho GTPase activating protein 25	X		X
MBP	Myelin basic protein	X		X
JMJD3	jumonji domain containing 3	X		X

In Tables 1 and 2, these transcripts have a predicted novel role in either untreated or treated RA, are not directly related to TNFα biology and have statistically significant impacts on DAS28 or joint health (denoted by X, *p*<0.05).

### Model intervention simulations – Virtual clinical trials

Systematic *in silico* simulations of 10-fold knockdown of all 9,306 transcripts were completed to provide quantitative predictions of how modulation of a particular gene expression measure would affect the DAS28 score in every particular subject given their own, individualized genotype and gene expression values before and after treatment with TNF-α blocker. Simulated distributions of SJ, TJ, Pain and CRP were compiled from 30 replicate gene expression simulations for each subject and for each gene. A single predicted DAS28 component score for each individual patient was then estimated as the median of the 30 replicate simulations to provide a robust point estimate of the range of predicted values. The simulated and summarized SJ, TJ, Pain and CRP scores were used to estimate a simulated DAS28 score using the standard equation supplied to clinicians.

Gene expression perturbations were ranked by their ability to sufficiently modulate RA clinical measures in a significant number of patients using either a χ^2^ test for TJ and SJ scores or a Student's t-test for Pain and CRP with respect to simulated untreated gene expression DAS28 scores.

### Identification of three classes of causal transcripts

78 transcripts from the untreated network ensemble (see Table S1) and 97 transcripts from the TNF-α blocker treated network ensemble (see Table S2) were predicted to significantly modulate any of the DAS28 component scores (*p*<0.05). The transcripts identified can be assigned to one of three broad categories based on their predicted efficacies and druggability, which was assessed using Ingenuity Pathway Analysis (Ingenuity Systems, www.ingenuity.com). Category 1 transcripts are defined as transcripts that have a predicted novel role in RA (Tables 1 and 2), are not directly related to TNF-α biology and have statistically significant impacts on DAS28 and joint health. Category 2 transcripts are potential alternatives to TNF-α therapies and have known dependencies on TNF-α or are proteins that could be targeted with a small molecule and are predicted to modulate DAS28 and joint health (Tables 3 and 4). Category 3 transcripts are those that are predicted to impact joint health but not DAS28 across the subjects simulated (Tables 5 and 6).

**Table 2:** Category 1 Transcripts from the pre-treatment model.

Gene Symbol	Name	TJ	SJ	DAS28
DOK3	Docking protein 3	X		X
C15orf39	Chromosome 15 orf 39	X		X
FLOT2	Flotillin 2	X		X
RHOG	Ras homolog gene family, member G (rhoG)	X		X
NBPF1	neuroblastoma breakpoint family, member 1	X		X
GLT1D1	glycosyltransferase 1 domain containing 1	X		X
FLJ43663	hypothetical protein FLJ43663		X	X

**Table 3:** Category 2 Transcripts from the post-treatment model.

Gene Symbol	Name	TJ	SJ	DAS28
TRAF3IP3	TRAF3 interacting protein 3	X		X
PSMD4	proteasome (prosome, macropain) 26S subunit, non-ATPase, 4	X		X
CD86	CD86 molecule	X	X	X

In Tables 3 and 4, transcripts are potential alternatives to TNF-α therapies and have known dependencies on TNF-α or are proteins that could be targeted with a small molecule and their predicted modulation might be expected to modulate DAS28 or joint health (*p*<0.05).

**Table 4:** Category 2 Transcripts from the pre-treatment model.

Gene Symbol	Name	TJ	SJ	DAS28
WARS	tryptophanyl-tRNA synthetase	X		X
LASS5	LAG1 homolog, ceramide synthase 5		X	X
CTSC	Cathepsin C		X	X

**Table 5:** Category 3 Transcripts from the post-treatment model.

Gene Symbol	Name	TJ	SJ	DAS28
1558906_a_at	NA	X	X	
MGST3	microsomal glutathione S-transferase 3	X		
FLJ22662	hypothetical protein FLJ22662	X	X	
LANCL1	LanC lantibiotic synthetase component C-like 1 (bacterial)	X		
SFRS18	splicing factor, arginine/serine-rich 18	X	X	
KPNB1	karyopherin (importin) beta 1	X	X	
EXOSC6	exosome component 6	X		
RAD23A	RAD23 homolog A (S. cerevisiae)	X		
DPYD	dihydropyrimidine dehydrogenase	X	X	
FUSIP1	FUS interacting protein (serine/arginine-rich) 1	X	X	
WIPF2	WAS/WASL interacting protein family, member 2	X	X	
CFLAR	CASP8 and FADD-like apoptosis regulator	X		
HNRPA3P1	heterogeneous nuclear ribonucleoprotein A3 pseudogene 1	X		
INHBC	inhibin, beta C	X	X	
COX18	COX18 cytochrome c oxidase assembly homolog (S. cerevisiae)	X		
FGL2	fibrinogen-like 2		X	
GSTO1	glutathione S-transferase omega 1		X	
FLJ43663	hypothetical protein FLJ43663		X	
TNRC6B	trinucleotide repeat containing 6B		X	
EIF3F	eukaryotic translation initiation factor 3, subunit F		X	
MBNL1	muscleblind-like (Drosophila)		X	
SUB1	SUB1 homolog (S. cerevisiae)		X	
PRDM2	PR domain containing 2, with ZNF domain		X	
C10orf46	chromosome 10 open reading frame 46		X	
CENTB2	centaurin, beta 2		X	
LRRFIP2	leucine rich repeat (in FLII) interacting protein 2		X	
DOCK8	dedicator of cytokinesis 8		X	
TMEM14C	transmembrane protein 14C		X	
OSGEP	O-sialoglycoprotein endopeptidase		X	
ATP6AP2	ATPase, H+ transporting, lysosomal accessory protein 2		X	
LOC727918	hypothetical protein LOC727918		X	
RPS2	ribosomal protein S2		X	
VAPA	VAMP (vesicle-associated membrane protein)-associated protein A, 33kDa		X	
RNF6	ring finger protein (C3H2C3 type) 6		X	
HLA-DPB1	major histocompatibility complex, class II, DP beta 1		X	
EXT1	exostoses (multiple) 1		X	

In Tables 5 and 6, transcripts are predicted to impact joint health (*p*<0.05) but not DAS28 (*p*>0.05).

**Table 6:** Category 3 Transcripts from the pre-treatment model.

Gene Symbol	Name	TJ	SJ	DAS28
PITPNA	phosphatidylinositol transfer protein, alpha	X		
N4BP1	Nedd4 binding protein 1	X		
XPO6	exportin 6	X		
IMPDH1	IMP (inosine monophosphate) dehydrogenase 1	X		
HLA-E	major histocompatibility complex, class I, E	X		
DPYSL2	dihydropyrimidinase-like 2	X		
LRP10	low density lipoprotein receptor-related protein 10	X		
EIF4G2	eukaryotic translation initiation factor 4 gamma, 2	X		
RALY	RNA binding protein, autoantigenic (hnRNP-associated with lethal yellow homolog (mouse))	X		
NT5C2	5′-nucleotidase, cytosolic II		X	
PSMB9	proteasome (prosome, macropain) subunit, beta type, 9 (large multifunctional peptidase 2)		X	
STAT3	signal transducer and activator of transcription 3 (acute-phase response factor)		X	
FNBP1	formin binding protein 1		X	
IL32	interleukin 32		X	
TRBC1	T-cell receptor beta constant 1		X	
PCSK7	proprotein convertase subtilisin/kexin type 7		X	
SSH2	slingshot homolog 2 (Drosophila)		X	
MAPKAP1	mitogen-activated protein kinase associated protein 1		X	
LAMP2	lysosomal-associated membrane protein 2		X	
PCGF3	polycomb group ring finger 3		X	
RUNX1	runt-related transcription factor 1 (acute myeloid leukemia 1; aml1 oncogene)		X	
NFIL3	nuclear factor, interleukin 3 regulated		X	
CFLAR	CASP8 and FADD-like apoptosis regulator		X	

Transcripts predicted to impact joint health (*p*<0.05) but not DAS28 (*p*>0.05)

### Literature analysis of REFS identified intervention points

Intervention points identified by REFS were analyzed to identify significantly enriched biological process using the GOstats package from R/Bioconductor, an open source software for bioinformatics [27], [28]. Molecular processes associated with nuclear factor kappa-B (NF-kappa-B) were identified from the pretreatment gene list (Table 7). NF-kappa-B is a family of transcription factors that are induced by TNF-α and other stimuli, and are critically important for inflammatory processes [29]. Genes associated with myeloid cell differentiation are also enriched in the untreated samples. Myeloid cells, including neutrophils, monocytes and macrophages, are producers of TNF-α. These cells also express TNF receptors, and on stimulation with TNF-α can produce even more of this cytokine as well as other pro-inflammatory molecules [30]. Terms relating to T helper cell differentiation and IL-4 biosynthesis were also among the significantly enriched molecular processes for transcripts identified as important for RA under TNF-α blocker therapy (Table 8), perhaps reflecting T-cell related pathways as well as B-cell activations and affecting RA while TNF-α signaling is therapeutically suppressed.

**Table 7:** Significantly enriched immune response related GO terms for transcripts from untreated model.

GOBPID	Pvalue	Count	Size	Term
GO:0045639	0.000201651	2	5	positive regulation of myeloid cell differentiation
GO:0006955	0.002364924	10	778	immune response
GO:0045637	0.002648076	2	17	regulation of myeloid cell differentiation
GO:0030097	0.008668954	3	93	hemopoiesis
GO:0006436	0.009086186	1	2	tryptophanyl-tRNA aminoacylation
GO:0042386	0.035861484	1	8	hemocyte differentiation (sensu Arthropoda)
GO:0000086	0.044627838	1	10	G2/M transition of mitotic cell cycle
GO:0042345	0.048981641	1	11	regulation of NF-kappaB import into nucleus
GO:0043123	0.049036663	2	78	positive regulation of I-kappaB kinase/NF-kappaB cascade

**Table 8:** Significantly enriched immune response related GO terms for transcripts from anti-TNF-α treated model.

GOBPID	Pvalue	Count	Size	Term
GO:0045064	0.010795911	1	2	T-helper 2 cell differentiation
GO:0042097	0.010795911	1	2	interleukin-4 biosynthesis
GO:0042109	0.010795911	1	2	tumor necrosis factor-beta biosynthesis
GO:0045624	0.016150771	1	3	positive regulation of T-helper cell differentiation
GO:0030154	0.025370559	7	537	cell differentiation
GO:0042092	0.02677505	1	5	T-helper 2 type immune response
GO:0042093	0.02677505	1	5	T-helper cell differentiation
GO:0045086	0.032044769	1	6	positive regulation of interleukin-2 biosynthesis
GO:0045621	0.037286403	1	7	positive regulation of lymphocyte differentiation

### Network analysis predicts *LASS5* and *IL32* as TNF-α dependent causal factors for swollen joint count

*LASS5*, longevity assurance homolog 5, is identified as a modulator for number of swollen joints. It is a ceramide synthase that synthesizes ceramide *de novo*. It has been shown that overgrowth of rheumatoid synoviocytes leads to joint destruction, and ceramide regulates cell growth by inhibiting pro-survival signals such as those from *AKT*, *MEK* and *ERK* [31]. Ceramide is also known as a pro-inflammatory signaling molecule and may regulate several matrix metalloproteases that can degrade cartilage tissue [32], [33].

**Figure 3:**
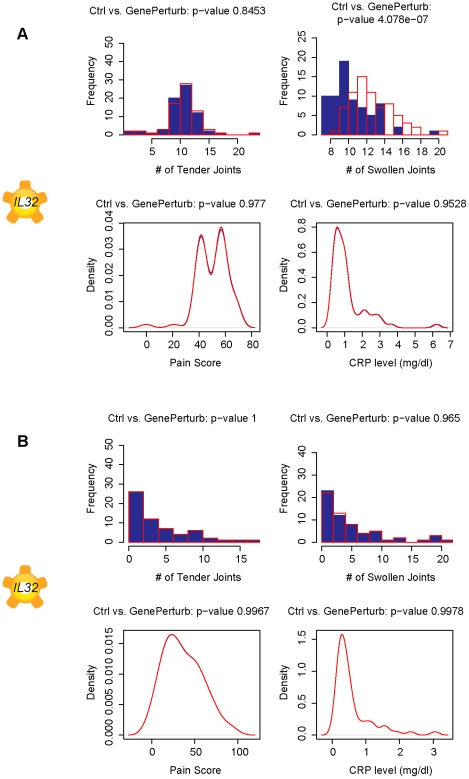
10-fold knockdown of *IL32* modulates number of swollen joints. Plots of simulated number of tender joints, swollen joints, pain and C-reactive protein concentrations in response to a 10-fold knockdown in gene expression of cytokine *IL32* in A. pretreated subjects and B. TNF-α blocker treated subjects. The effects are only predicted in pre-treated patients, suggesting a dependence on TNF-α signaling. The largest predicted effect is to modulate the number of swollen joints.

**Figure 4:**
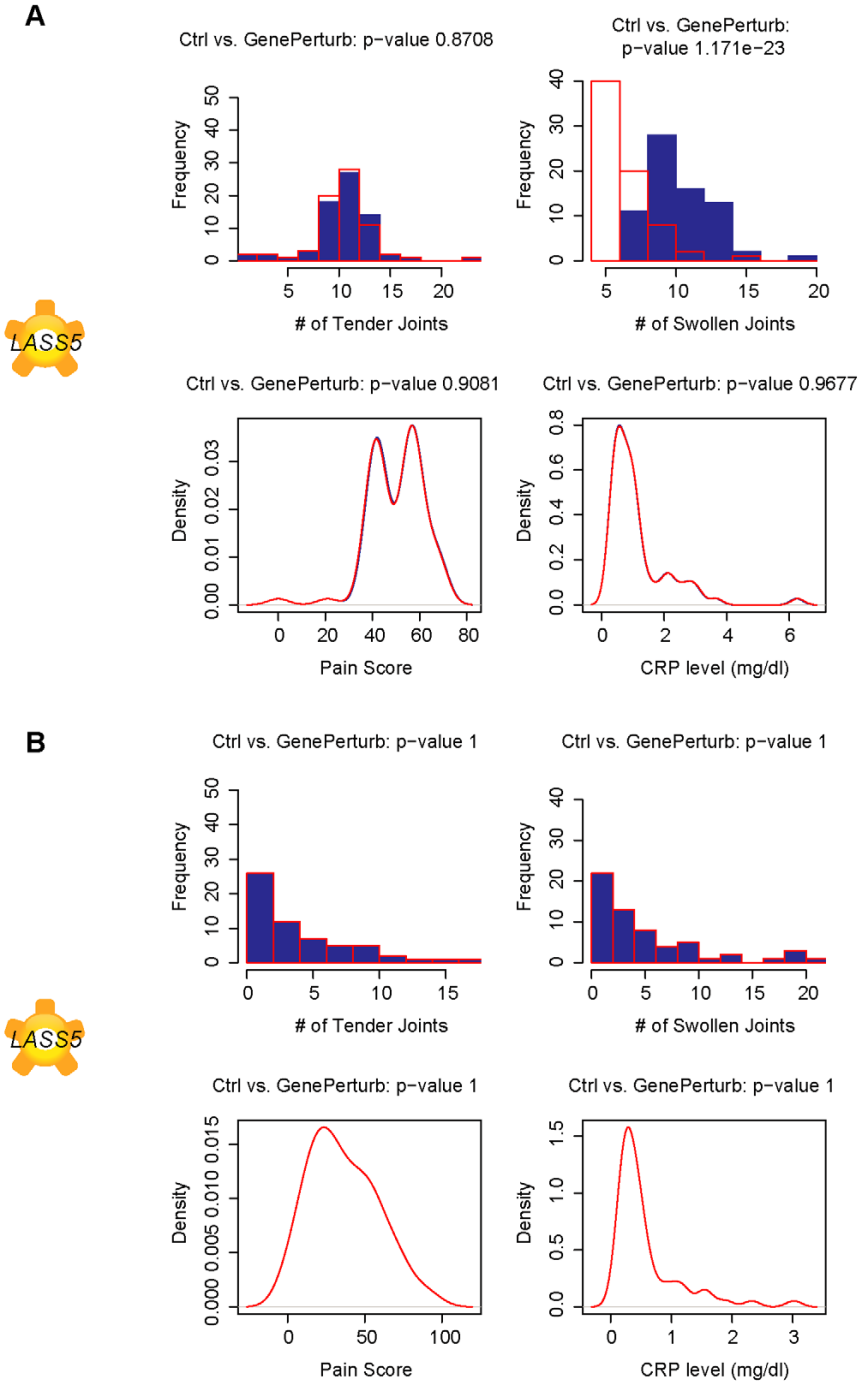
Swollen joints predicted to be modulated by *LASS5* in pre-treated patients only. Plots of simulated number of tender joints, swollen joints, pain and C-reactive protein concentrations in response to a 10-fold knockdown in gene expression of ceramide synthase, *LASS5*, in A. pretreated subjects and B. TNF-α blocker treated subjects. The modulation of swollen joints is only predicted in pre-treated patients, suggesting a dependence on TNF-α signaling.

Simulation results suggest that *LASS5* expression interacts with the expression of interleukin 32 (*IL32*) to modulate the number of swollen joints (Figure 3–4). *In silico* perturbations of *IL32* are predicted to affect number of swollen joints. *IL32* is a cytokine induced by TNF-α and may play a critical role in rheumatoid arthritis. Injection of human IL-32 protein into knee joints of mice leads to joint swelling as well as inflammation and cartilage damage and this effect is dependent on the presence of TNF-α [34]. The predicted effect of *IL32* is not detected in the network ensemble built from data from TNF-α blocker treated subjects, agreeing with experimental data and further suggesting that the treated network ensemble is capturing TNF-α independent mechanisms in RA.

### Network analysis predicts *WARS* as a TNF-α dependent causal factor for tender joint count

Persistence of autoimmune activated T-cells is a feature of RA [35]. Typically, T-cells can be suppressed by indoleamine 2,3-doxygenase (IDO) signaling which triggers the catabolism of tryptophan and subsequent suppression of T-cell response [36]. *WARS* is a tryptophanyl-tRNA synthetase whose gene expression is significantly elevated in T-cells derived from the synovial fluid of RA patients and leads directly to the sequestration of intra-cellular tryptophan in the form of tryptophanyl-tRNA. With intra-cellular stores of free tryptophan lowered by over-expression of *WARS*, IDO signaling is now muted and can explain the persistence of activated T-cells in RA patients and their resistance to IDO [37]. Simulations suggest that knockdown of *WARS* gene expression can significantly affect the number of tender joints sufficiently and strongly enough to modulate DAS28.

**Figure 5:**
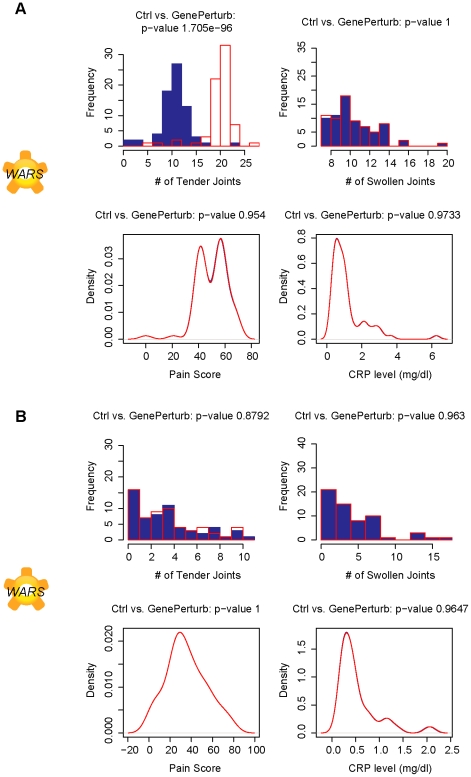
Tender joints predicted to be modulated by *WARS* in pre-treated patients only. Plots of simulated number of tender joints, swollen joints, pain and C-reactive protein concentrations in response to a 10-fold knockdown in gene expression of tryptophanyl-tRNA synthetase, *WARS*, in A. pretreated subjects and B. TNF-α blocker treated subjects. The modulation of tender joints is only predicted in pre-treated patients, suggesting a dependence on TNF-α signaling.

*WARS* gene expression is predicted to be a modulator of RA only in pretreated subjects, suggesting that *WARS* mechanism is TNF-α dependent (Figure 5). This assertion is confirmed because enhanced gene expression of *WARS* that leads to tryptophan sequestration is dependent on TNF-α in experimental systems [37].

### *CD86* is a predicted to be a TNF-α independent causal factor for joint health

**Figure 6:**
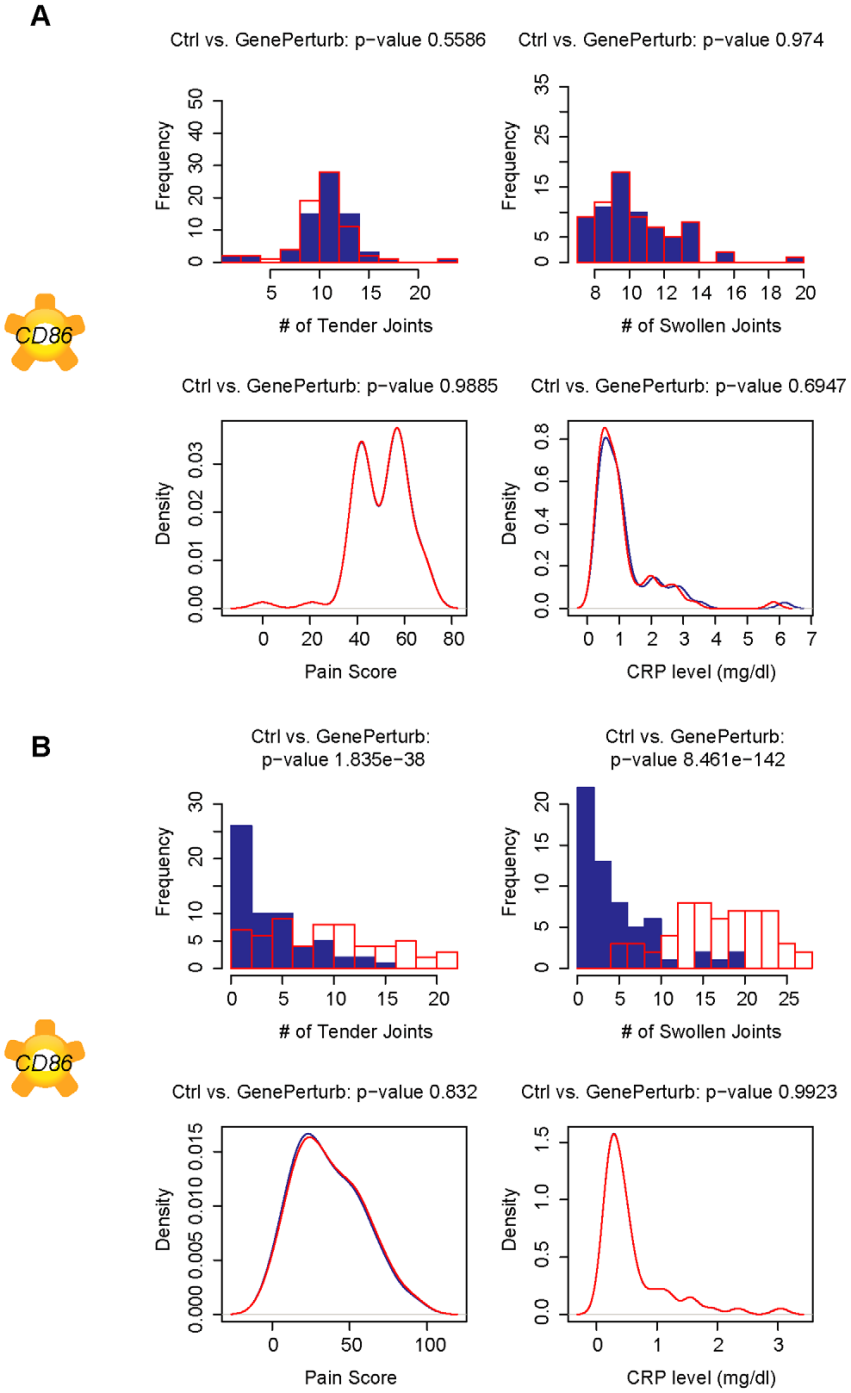
Modulation *CD86* predicted to affect both tender and swollen joint counts in TNF-α treated patients. Plots of simulated number of tender joints, swollen joints, pain and C-reactive protein concentrations in response to a 10-fold knockdown in gene expression of *CD86*, the target of abatacept (CTLA4-Ig), in A. pretreated subjects and B. TNF-α blocker treated subjects. The modulation of tender joints is only predicted in TNF-α treated patients, suggesting both a mechanism that is independent of TNF-α signaling that could be exploited for subjects that do not respond well to TNF-α blocker therapies.

*CD86* (B7-2) is identified as a strong modulator of TJ, SJ and DAS28 in the network analysis of TNF-α blocker treated patients and should represent a particular TNF-α independent mechanism that is active in RA patients (Figure 6).

*CD86* is a type I membrane protein expressed by antigen-presenting cells (APC) and is the ligand for *CD28* and *CTLA4* on the surface of T-cells. Binding of this ligand to *CD28* or *CTLA4* provides co-stimulatory signals that can either positively or negatively regulate T-cell activation that are independent of TNF-α.

Abatacept (CTLA4-Ig) is an approved biologic drug that exploits this mechanism by blocking the co-stimulatory signal from CD80/CD86 thus preventing the full activation of T-cells [38]. The drug is approved for patients that have shown unsatisfactory response to TNF-α blocker drugs, clearly demonstrating that network reconstruction and simulation of data collected under TNF-α blocker treatment recovers TNF-α independent mechanisms that could be used to identify drug targets for the segment of the population that does not respond to TNF-α blocker therapy.

### *RAP2C* and *GON4L* are novel predicted TNF-α independent causal factors for TJ and SJ

**Figure 7:**
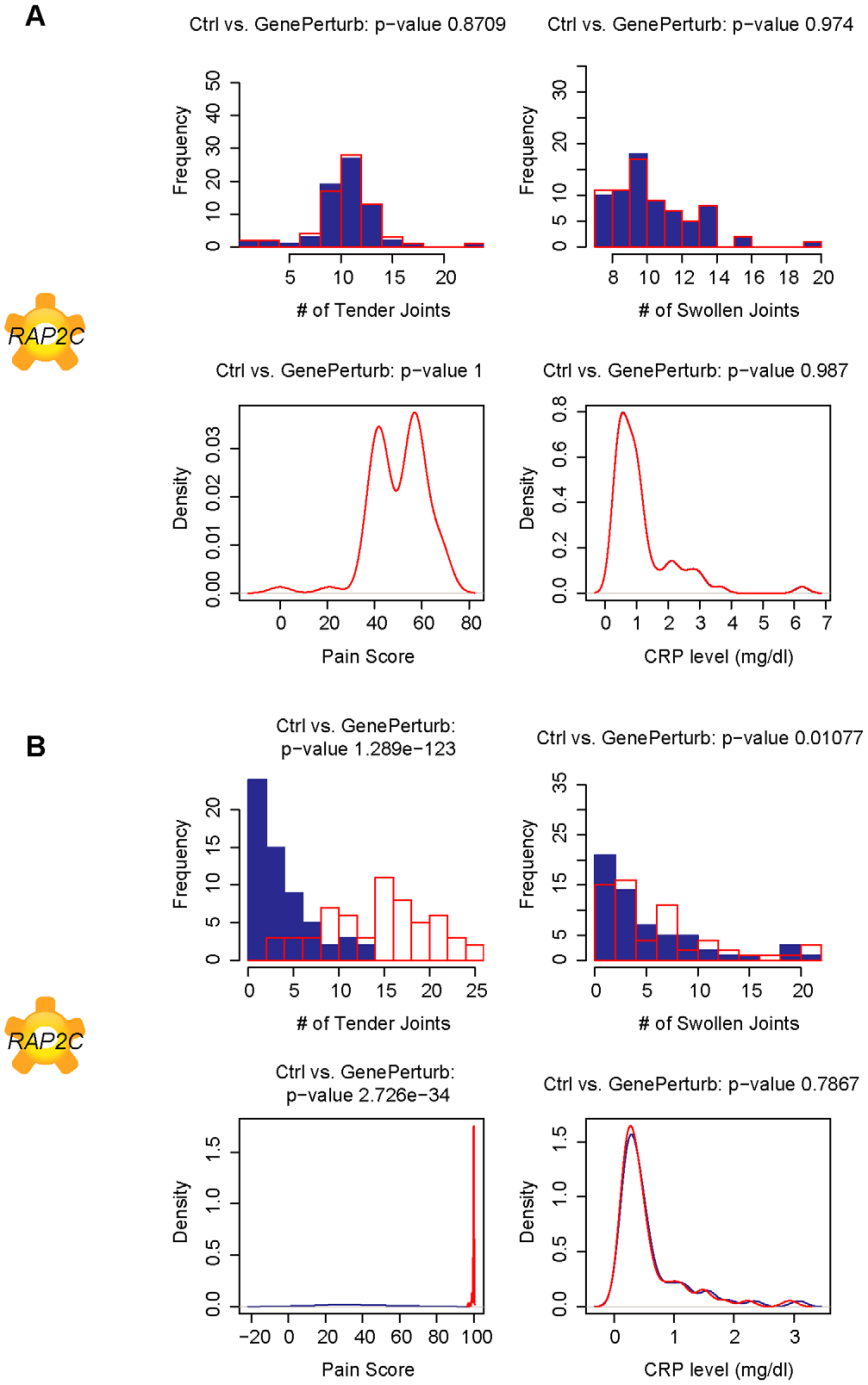
*RAP2C* predicted to modulate both tender and swollen joint counts in TNF-α treated patients. Plots of simulated number of tender joints, swollen joints, pain and C-reactive protein concentrations in response to a 10-fold knockdown in gene expression of *RAP2C*, a recently described ras G-protein, in A. pretreated subjects and B. TNF-α blocker treated subjects. The simulations suggest that this novel gene can modulate both tender and swollen joint count in TNF-α treated subjects. *RAP2C* may provide insight into novel TNF-α independent signaling pathways in RA.

**Figure 8:**
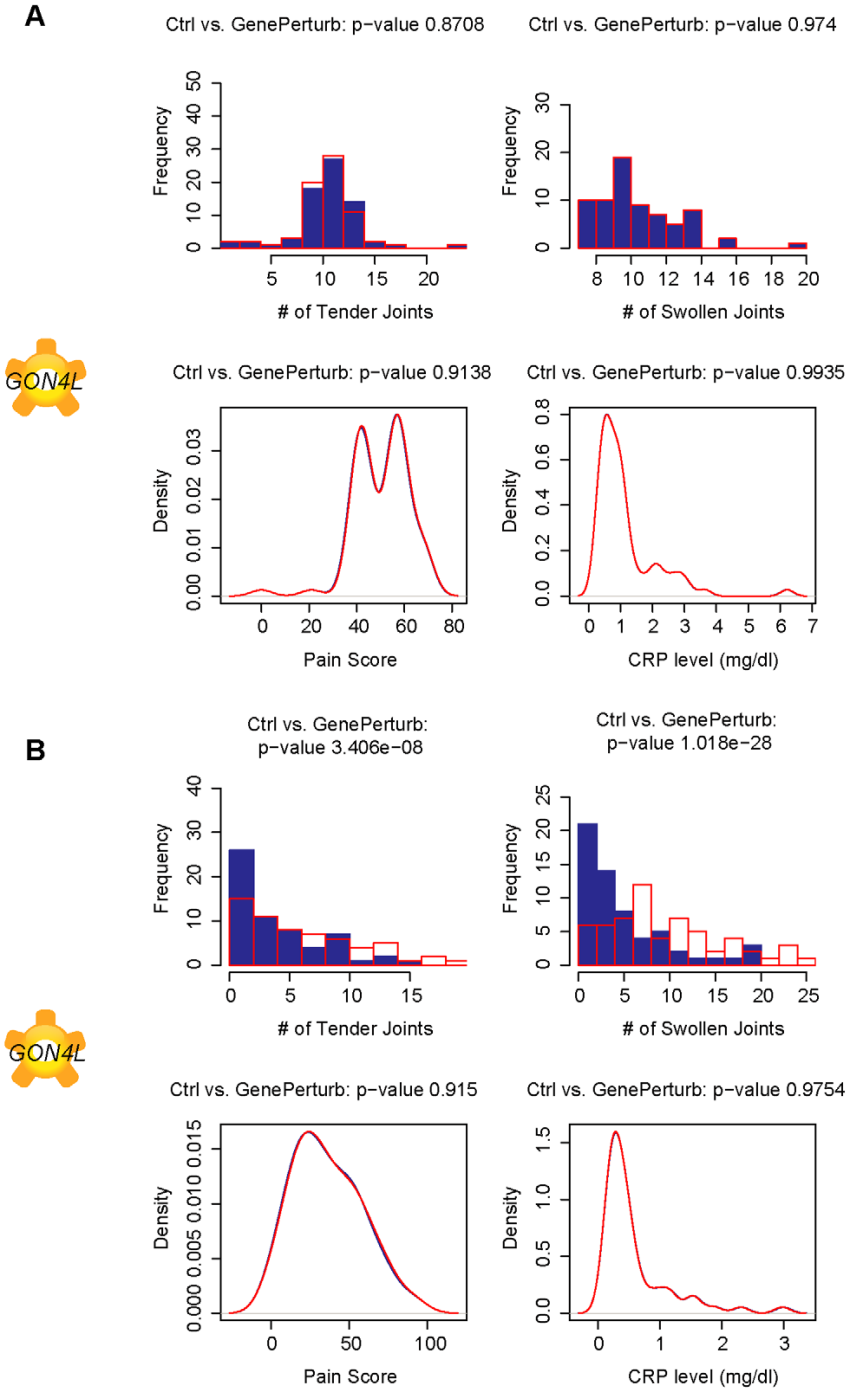
*GON4L* predicted modulate both tender and swollen joint count in TNF-α treated patients. Plots of simulated number of tender joints, swollen joints, pain and C-reactive protein concentrations in response to a 10-fold knockdown in gene expression of *GON4L*, recently described as a novel factor in B-cell differentiation, in A. pretreated subjects and B. TNF-α blocker treated subjects. The simulations suggest that this novel gene can modulate both tender and swollen joint count in TNF-α treated subjects. *GON4L* may provide insight into novel TNF-α independent signaling pathways in RA.

*In silico* perturbations further identified *RAP2C* (Figure 7) and *GON4L* (Figure 8) as causal factors for TJ, SJ and DAS28 score. Very little is currently known about either of these transcripts and this is the first analysis that suggests a role for these transcripts as TNF-α independent modulators of joint health in RA patients.

*RAP2C* is a novel member of Ras G-protein family and the model predicts that perturbation of *RAP2C* leads to significant changes of TJ, SJ and DAS28 score.

*GON4L* is a human ortholog of the *Caenorhabditis elegans* cell lineage regulator of gonadogenesis *GON4L*. This gene is a putative transcription factor that may regulate cell cycle control. *GON4L* has been shown to be an essential regulator of B-cell development [39]. Notably, B-cells are the target for rituximab, a monoclonal antibody against CD20 that is approved for the treatment of RA patients with insufficient response to TNF-α blockade [40]. B cells produce IL-6 upon activation, and IL-6 blocking molecules have also been approved for RA treatment [41]. Our analysis suggests that *GON4L* may be a novel target for RA by modulating B-cell differentiation.

## Discussion

It is important to recognize that the network ensembles are built from gene expression profiles of whole blood and are not directly measured from diseased tissue in the joints, which complicates the assessment of the transcripts identified as outright targets in RA. Additionally, mRNA from whole blood had to be depleted of hemoglobin mRNA and the resulting subtracted mRNA sample was amplified using specialized PCR fluorescence labeling protocols that may introduce bias in the measurements. The unavoidable technical details meant that collected data were noisy and there was a large amount of network structure diversity recovered in the ensemble sample after network reconstruction. However, the predicted magnitude of the effects of some of the transcripts identified was large enough to infer significant effects despite the uncertainty in the network ensemble. Furthermore, when interpreting inferences from the network ensemble it is important to recognize that there could be hidden common or intermediate causes that were not part of the measured dataset that could modify the logic of the causal inferences encoded in the ensemble.

The transcripts identified using network simulations represent quantitative hypotheses about control points in untreated and TNF-α blocker treated RA patients. The promise of this approach is exemplified by the recognition of the T cell co-stimulatory molecule CD86 and the B-cell restricted molecule GON4L in the TNF-α blocker treated samples. The network simulations were based purely on the data and did not include any up-front literature information or supervision. The models suggest that modulation of these molecules would impact disease scores, and these molecules represent previously validated pathways for treating patients with insufficient response to TNF-α blockade. Not every intervention point identified by the models will be as clear as CD86, and the genes we identify should be considered as starting points in the investigation of a wider range of pathway and signaling mechanisms that have not been widely examined for therapeutic benefit. For example, the therapeutic potential of a systematic *WARS* inhibitor is questionable but other molecular components of IDO signaling might present better therapeutic potential.

Rheumatoid Arthritis drugs recommended by The American College of Rheumatology (ACR) fall into several categories: small molecule DMARDs including methotrexate, lefluonamide and others; anti-TNF-α agents; T-cell activation modulators; IL-6 antagonists; IL-1 antagonists; and B-cell directed therapy. The analysis conducted in this study is relevant for all of these therapeutic approaches, but ideally suited for identifying new pathways to target in patients with insufficient response to first-line DMARDS and biologics such as anti-TNFs. Whereas the DMARDS have relatively broad mechanisms of action impacting several inflammatory pathways, the biologics all have very targeted mechanisms and are generally used only after DMARDS have failed. While the pleiotropic effects of the biologics overlap, each class of biologics targets a specific feature of inflammation. The anti-TNFs are by far the most common, and these target inflammatory processes driven by macrophages that are close to the top of the inflammatory cascade. In current practice, other biologics are used primarily after DMARDS and anti-TNFs fail. This partly reflects the culture of new drug adoption – anti-TNFs have a track record and there are several to choose from – but may also reflect underlying disease heterogeneity. The network analysis supports the notion that underlying disease heterogeneity is important. Different biological pathways are active with and without anti-TNF-α treatment. Furthermore, T-cell and B-cell pathways targeted by approved RA drugs clearly emerge from the model as reflected by the impact of modulating CD86, part of the abatacept (CTLA4-Ig) target, and GON4L, expressed in B cells that are the target of rituximab (anti-CD20).

Candidate targets identified by network analysis can be put into specific inflammatory pathways. While the pathways have been previously identified, some of the specific transcripts have not been recognized as being associated with RA before. These newly identified transcripts highlight pathways for small-molecule or biologic treatments of RA. In addition to the few genes mentioned above, network analysis predicts additional transcripts such as *STAT3*. STAT3 has been identified as a pro-survival molecule for RA synoviocytes [42], and has also been shown to mediate some of the pro-inflammatory signaling of IL-6 {Hirano, #88} – yet another target of approved RA treatments. Additionally, *FLJ43663* is predicted to be a secreted protein and represents a novel type of molecule identified through network simulation.

Of the eighteen genes significantly affecting counts of Swollen Joints (SJ), correlative statistical models identified only two transcripts, *CTSC* and *IL32*. REFS identified the importance of *RUNX1*, a transcription factor that regulates genes such as *BLK*, *TCR*, *CD3* and *GM*-*CSF* in lymphoid cells [44] and which may play a role in autoimmune disease such as rheumatoid arthritis [45]. REFS ranked *RUNX1* as the fifth most important transcript modulating SJ while correlative statistical models ranked *RUNX1* as the 1280^th^ most important transcript (See Figure S4A). Further investigation of the REFS network model suggests that *RUNX1* affects SJ by modulating hypothetical gene *FLJ43663*. In this manner, REFS identified a completely novel intervention point and provided insight into its potential mechanism (See Figure S4B).

Network models that incorporate genetic, molecular, and clinical data collected from longitudinal samples represent a powerful complement to classical statistical models for identifying genes and pathways important for disease processes. We developed ensemble models for a small cohort of RA patients sampled prior to and during treatment with TNF-α blockers. These ensemble models accurately predict the involvement of known RA drug targets including T cell co-stimulation and B cell regulation. These observations suggest that other genes and pathways identified in the network ensembles represent promising targets for further investigation.

## Methods

### Processing and imputation of genotyping data

The Illumina HAP300 chip was used to profile the genotypes of patients. The most recent genomic coordinates for human SNPs were downloaded from the Ensembl website. The updated genomic positions were used together with MACH [46], a Markov-chain haplotyper to impute missing genotypes from the data as well as previously identified SNPs associated with RA. HLA types were mapped to SNP positions based on HLA-SNP map [47]. All genotypes from the X and Y chromosome were removed from consideration because the analysis of hemizygous genotypes produces false positive associations. SNPs that failed Hardy-Weinberg equilibrium test, or had a minor allele frequency (MAF) (*p*<0.05), or had a call-rate<95% were also removed. The SNP QC process resulted in 279,557 SNPs selected for further analysis and the process is detailed in Table 2 in Text S1.

EIGENSTRAT was used to detect and correct for population stratification on a genome-wide scale that was detected in the full data set using smartPCA. The associated Armitage chi-squared statistic is computed for each SNP and used to rank SNPs associated with phenotypes to produce a ranked list of SNPs that were potentially important in explaining RA treated and untreated phenotypes. 6,075 SNPs and 6,076 SNPs were chosen from the ranked lists to model untreated and treated subjects respectively.

### Assessment and processing of gene expression data

Simpleaffy was used to assess the quality of microarray hybridization [48]. The majority of samples lay within the tolerances suggested by Affymetrix for amplified RNA samples. The entire microarray data set was normalized using FARMS [49]. This normalization technique has outperformed previously developed methods in the Affycomp II competition [50] in detecting differential gene expression and is used in conjunction with an associated package called I/NI (Informative/Non-Informative) that uses variance across the dataset to identify informative genes for further analysis [51].

### Assessment and processing of DAS28 data

All subjects in the data had DAS28 scores for all visits and the components of the DAS28 score were also available. Pair plots of the components of the DAS28 scores show that the components themselves are orthogonal to each other and capture different aspects of RA. Visual analogue scale for overall health assessment scores and the health assessment questionnaire showed a high degree of correlation with the pain score component of the DAS28 and were considered redundant.

Tender joint count (TJ), Swollen joint count (SJ) and Pain scores were logit transformed with an additional discrete to continuous continuity correction applied prior to REFS modeling. C-reactive protein concentrations (CRP) were log transformed prior to analysis to ensure valid, non-zero simulation results in response to intervention queries and to stabilize the variance across the data set.

### Learning probabilistic models from data

A multivariate system with random variables *X = (X_1_, …, X_n_)* where each variable may take on values from a discrete (genetic markers) or continuous domain (gene expression and phenotypic data) may be characterized probabilistically by a joint multivariate probability distribution function *P(X_1_, …, X_n_; Θ).* However, full specification of such joint probability distributions requires a large number of parameters *Θ*. Such a global joint probability distribution admits the following factorization into a product of *local* conditional probability distributions:
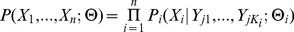
(1)where each variable *X_i_* is independent of its nondescendants given its *K_i_* parents *Y_j1_, …, Y_jKi_* (local Markov condition) and *Θ_i_* are parameters for *P_i_*. The *Y* variables are simply a subset of the *X*'s; we use the *Y* notation to indicate they are inputs to the conditional probability. This approach yields a framework where each particular factorization and choice of parameters is a distinct probabilistic model *M* of the structure of the process that created the observed data [11]. Learning these models *M* from a data set *D* is simply determining which factorizations of *P(X_1_, …, X_n_;Θ)* are most likely given the observation of *D*, and given a factorization, what are the likely values for its parameters *Θ = (Θ_1_, …, Θ_n_).*

Each factorization of *P(X_1_, …, X_n_)* into model *M* (as in Eq. 1) is represented by a unique Directed Acyclic Graph (DAG) *G* with a vertex for each *Xi* and directed edges between vertices to represent the dependencies between variables embedded in the local conditional distributions, *P_i_(X_i_|Y_j1_, …, Y_jKi_)*. In addition to the graph *G*, *M* also specifies distributions for all *Θ_i_* the parameters of local conditional distributions *P_i_*. Subgraphs of *G*, consisting of a vertex and a set of all its incoming edges, and associated local conditional distributions *P_i_* and parameters *Θ_i_*, are referred to here as “network fragments”. We interpret each of these network fragments *M_i_* to characterize both the functional variation of its output variable *X_i_* with respect to its parent input variables *Y_j1_, …, Y_jKi_* and the residual variation in *X_i_*. For integrative genomics we consider several specific functional forms for network fragments and used linear regression. First, consider the case where all of the input variables *Y_j1_, …, Y_jKi_* are continuous, then we model the centroid of *X_i_* by: 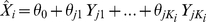
(2)and *X_i_* by a normal distribution about that value:
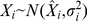
(3)The parameters σ*_i_, θ_0_, θ_j1_, …, θ_jKi_* can be thought of as adjusted to best fit the data in the Maximum Likelihood Estimation (MLE) sense. The likelihood function gives the posterior distribution of the parameter values about the MLE point. Next, consider the case where one of the *Y* variables is discrete. To model its influence its linear term in Eq. 2 is dropped and the discrete value is used to switch the value of the remaining linear fitting parameters. That is to say, for each value of the discrete variable a *different* set *θ_0_, θ_j1_,* …, *θ_jKi_* of fitting parameters is introduced. Finally, if multiple *Y*'s are discrete all of their linear terms are dropped from Eq. 2 and their joint discrete state is used as the switching value. In this study all of the output variables *X_i_* are continuous: discrete variables are only taken as inputs.

### Parallel ensemble sampling

To determine which factorizations are likely given the data we use a Bayesian framework to compute the posterior probability of the model *P(M|D)* from Bayes' Law
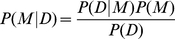
(4)where *P(D)* is the probability of *D, P(M)* is the prior probability of the model and 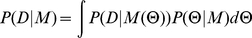
(5)is the integral of the data likelihood over the prior distribution of parameters Θ. We assume that data is complete. Assuming that parameters *Θ* are independent, all models are equally likely, and *P(D)* is constant, we factor *P(M|D)* in Eq. 4 into the product of integrals over the parameters local to each network fragment *M_i_*_._ Eq. 4 now becomes
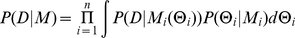
(6)where *P*(Θ*_i_* |*M_i_*) is the network specific prior for its parameters.

For this work we use Schwartz's Bayesian Information Criterion approximation to the above integral (asymptotically exact as the number of samples increases):
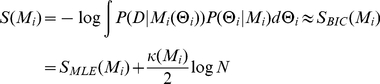
(7)where κ(*M_i_*) is the number of fitting parameters in model *M_i_* and *N* is the number of samples. We refer to *S* as a “score”, but note the minus sign in the definition (to agree with the simulated annealing analogy described below) and so lower scores are more likely. *S_MLE_* is the negative logarithm of the MLE value of the likelihood function.

The total network score is:
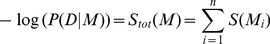
(8)a sum over the scores of each network fragment in the candidate graph model. In principal the repository of candidate network fragments can be constructed by exhaustive enumeration over variables and network fragment forms. We selected models that provided highest likelihood [52], [53] and considering at most 2 edges for a particular vertex. However, even with these constraints, the space of all possible graphs is still too large to be sampled by exhaustive enumeration.

Instead we use the Metropolis method (Markov Chain Monte Carlo) to generate samples from an equilibrium Boltzmann distribution of candidate structures [54] from *P(M|D)*. Each step in a Metropolis Markov Chain corresponds to local transformations such as adding or deleting network fragments. To accelerate convergence we used simulating annealing were we applied the Metropolis method to a sequence of distributions 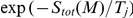 with decreasing *T_j_* (annealing temperature). At each stage *j* the equilibrated samples from *T_j_* initialize the Metropolis method at *T_j+1_*. Convergence of the random walk is monitored along the way and the annealing schedule is dynamically modified to take more Monte Carlo steps when the barriers present a larger obstacle to diffusion through the space of networks. The method for doing this was to estimate rate of change with respect to *T* of the mean total score 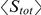 and also its variance 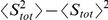 (the angle brackets denote Monte Carlo averages over networks at the current *T*.) From these values the change in temperature, 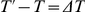, is selected so that the distribution of 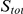 at 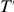 will have 80% overlap with the distribution at *T*. This process of maintaining overlap helps ensure that the sampling will be correct when *T = 1* is reached. In addition, shorter runs were performed to confirm that results are consistent with the longer runs.

In the normal usage of simulated annealing to find a global optimum, the control parameter *T* is allowed to go below 1; as long as better solutions are still being found the temperature is allowed to decrease. In our approach we stop at *T = 1* because the sampling there corresponds directly to the posterior distribution *P(M|D)* in Eq. 4; going to lower values of *T* would lead to over fitting the data.

### Model intervention simulations

Stochastic simulation of a probabilistic model *M* allows predictions about the distribution of a variable *Xi* to be made under different conditions. The conditions can be interventions with variables in the model and/or different values of inputs to the model. We used Gibbs sampling in which each variable *Xi* is sampled from its conditional Gaussian distribution, such as Eq. 2, 3, whose parameters take on most likely values given data *D*. For simulation of subjects not seen in the training data, only roots of the graph G had values. A simulation routine iteratively sweeps the network and generates samples of variables whose parents have already acquired a value in previous iterations until all variables have values. One full sweep produces one sample (one vector of values of all variables). Interventions such as a knockdown of gene transcript expression level variables are done by removal of the network fragment from M that outputs to the variable and the network is swept as described previously.

For each subject in the data analysis, the contribution of each gene simulation was assessed conditioned by the genotype and the other gene expression measures of the subject.

## Supporting Information

Figure S1 Predicted phenotypic values of training data for untreated model. Plot of simulated data for phenotypes (number of tender joints, number of swollen joints, pain score, and CRP levels) based on untreated model vs. observed values. Dashed line represents the line of unity (y = x) and each circle represents a patient in the training data. Correlation coefficients (r) for each phenotype are printed as part of the title.
(0.75 MB EPS)

Figure S2 Predicted phenotypic values of training data for treated model. Plot of simulated data for phenotypes (number of tender joints, number of swollen joints, pain score, and CRP levels) based on treated model vs. observed values. Dashed line represents the line of unity (y = x) and each circle represents a patient in the training data. Correlation coefficients (r) for each phenotype are printed as part of the title.
(0.74 MB EPS)

Figure S3 Predicted phenotypic values of test data. Plot of simulated data for phenotypes (number of tender joints, number of swollen joints, pain score, and CRP levels) based on treated model vs. observed values. Dashed line represents the line of unity (y = x) and each circle represents a patient in the test data. Correlation coefficients (r) for each phenotype are printed as part of the title.
(0.74 MB EPS)

Figure S4 Comparison of REFS with statistical models & Network topology of example gene. A. Comparison of genes identified by REFS™ and correlative statistical models for swollen joint (SJ) counts of untreated data. B. A Schematic representation of genes upstream of SJ from untreated model.
(3.03 MB EPS)

Table S1 Significant transcripts identified from untreated network ensemble. Transcripts from the untreated network ensemble were predicted to significantly modulate any of the DAS28 component scores (p<0.05).
(0.03 MB XLS)

Table S2 Significant transcripts identified from the TNF-α blocker treated network ensemble. Transcripts from theTNF-α blocker treated network ensemble were predicted to significantly modulate any of the DAS28 component scores (p<0.05).
(0.03 MB XLS)

Text S1 Assessment of the accuracy of the network Ensemble.
(0.06 MB DOC)
