# Supplementary material for: Causal Modeling Using Network Ensemble Simulations of Genetic and Gene Expression Data Predicts Genes Involved in Rheumatoid Arthritis
Source: PLoS Comput Biol. 2011 Mar 10;7(3):e1001105. doi: 10.1371/journal.pcbi.1001105 (PMC3053315; doi:10.1371/journal.pcbi.1001105)
Supplement: Text S1 — Assessment of the accuracy of the network Ensemble. (0.06 MB DOC) [file pcbi.1001105.s007.doc]

# Assessment of the accuracy of the network Ensemble

The accuracy of the model in learning the clinical data was assessed in two ways. First, the genetic and gene expression data used to train the data were used to initiate Monte Carlo sampling to recall the RA phenotypes in a data recall exercise. The plots of inferred RA phenotypes from the model, given the genetic and microarray data against the actual observed phenotypes for the TNF-alpha treated and naïve samples are given in Figure S1-S2.

Typically, cross-validation is used to assess the accuracy of the of algorithm’s predictions to extend to out-of-sample predictions and so give some measure of the generality of the predictions. Cross-validaton, however is an inherently biased method where a partition can be found in the data that maximizes the cross-validation reporting metrics and the process, including the process to select the informative set of variables should be repeated many times to give an accurate metric. Due to the complexity and running time of the entire algorithm and the biases in cross-validation methodology, it was neither desirable nor practical to complete a cross-validation assessment for both treated and untreated models.

Instead, a data set from ABCoN study was sourced for the patients after they had been exposed to TNF-alpha blocker for 6 weeks. At 6 weeks, some patients were beginning to experience modification of their RA symptoms, but their response was not yet fully evolved. This independent data collected at 6 weeks was used to assess the accuracy of the TNF-alpha treated network. If the network collected at 16 weeks describes a representation of the underlying probabilistic causes of RA symptoms while under treatment, then it is reasonable to expect the ensemble to predict partially treated RA symptoms given microarray data collected in this partially resolved state even though this was not used to train the ensemble.

Correlation and root mean squared error (RMSE) between the predicted 6-week RA phenotypes and observed RA phenotypes at 6 weeks were calculated (Table 1 in Text S1). The most clinically relevant and heavily weighted measures of the DAS28 score (see Methods for DAS28 calculation formula) are the number of tender joints and the number of swollen joints, with the self-reported pain score and C-Reactive Protein (CRP) measures having little weight.

Using the 6-week, partial response gene expression data to query the 16-week model resulted in correlation coefficient for the inferred number of tender joints against the observed number of 0.625 and RMSE of 1.07; and for the number of tender joints a correlation coefficient between the inferred number and the observed number of 0.442 and RMSE of 1.28, demonstrating that the network ensemble is making sensible inferences about the most important aspects of RA in out of sample data. The correlation coefficients for inferred Pain and CRP against observed Pain and CRP detailed in Table 1 in Text S1 show that the network ensemble is unlikely to be making accurate predictions about these RA symptoms. This is not unexpected as it is unlikely that transcription profile from whole blood would contain molecular information that could explain a self-reported pain scale score

**Table 1.** Statistical Evaluation of the performance of models based on predicting phenotypes of training data and test data.

|  | **Correlation Coefficient (r)** | | | | **RMSE** | | | |
| --- | --- | --- | --- | --- | --- | --- | --- | --- |
|  | **TJ** | **SJ** | **Pain** | **CRP** | **TJ** | **SJ** | **Pain** | **CRP** |
| **Training Data (untreated model)** | 0.894 | 0.872 | 0.772 | 0.862 | 0.770 | 0.477 | 0.799 | 0.648 |
| **Training Data (treated model)** | 0.828 | 0.872 | 0.849 | 0.841 | 0.937 | 0.782 | 0.883 | 0.532 |
| **Test Data (treated model)** | 0.625 | 0.442 | 0.0732 | 0.278 | 1.07 | 1.28 | 1.57 | 1.16 |

RMSE: root mean squared error, TJ: number of tender joints, SJ: number of swollen joints, Pain: pain score, CRP: plasma C-reactive protein level.

**Table 2.** SNP data processing pipeline.

| **Process** | **Number of SNPs** | **Number of Samples** |
| --- | --- | --- |
| Start point | 317,503 | 131 |
| Remove SNPs from X, Y chromosome | 308,330 | 131 |
| Remove patient with >5% missing genotype calls | 131 🡪 126 patients | 126 |
| Call rate per SNP (>95%) | 295,240 | 126 |
| Overlapping with Illumina 1M-Duo | 289,764 | 126 |
| Overlapping with Hapmap | 289,764 | 126 |
| Population stratification (Select European origins) | 126 🡪 104 patients | 104 |
| Genotype imputation | Using MACH | 104 |
| HWE (p>0.001) & MAF> 0.05 | 279,557 | 104 |
